# Linguistic Skills in Bilingual Children With Developmental Language Disorders: A Pilot Study

**DOI:** 10.3389/fpsyg.2019.00493

**Published:** 2019-03-07

**Authors:** Andrea Marini, Paola Sperindè, Isabella Ruta, Christian Savegnago, Francesco Avanzini

**Affiliations:** ^1^Department of Languages, Literatures, Communication, Education and Society, University of Udine, Udine, Italy; ^2^Claudiana – Landesfachhochschule für Gesundheitsberufe, Bolzano, Italy; ^3^Servizio di Neurologia e Riabilitazione Pediatrica, Ospedale di Bolzano, Bolzano, Italy; ^4^Medico Ditrigente di Foniatria, Reparto di Otorinolaringoiatria, Ospedale di Bolzano, Bolzano, Italy

**Keywords:** developmental language disorders, primary language impairment, specific language impairment, bilingualism, narrative discourse, BVL_4-12

## Abstract

The current pilot study compared the linguistic characteristics of a cohort of simultaneous bilingual children (Italian, L1; German L2) with developmental language disorders (DLDs) and those of bilingual peers with typical language development (TLD). Importantly, the two groups were balanced for a number of environmental variables (e.g., age of first exposure to the L2, acquisition contexts, degree of exposure to both languages) known to affect linguistic development in both TLD and DLDs. The analyses included the assessment of the participants’ phonological short-term memory. Their lexical, grammatical and narrative abilities were analyzed in both languages by administering the Italian and German equivalent forms of the *Battery for the assessment of language in children aged 4 to 12* – BVL_4-12 (Marini et al., 2015). The children with DLDs had reduced phonological short-term memory and lexical skills that, in turn, contributed to the reduced levels of local coherence and informativeness of their narratives. Such difficulties were found at similar levels in their two languages. These results suggest that reduced phonological short-term memory and lexical selection skills may reflect a core symptom in both mono- and bilingual children with developmental language disorders.

## Introduction

Recent estimates suggest that approximately 7,000 languages are currently spoken worldwide in barely 196 Countries (Simons and Fennig, 2018). This suggests that the vast majority of the world’s population is consistently exposed to two or more languages and can be considered bilingual (Baker, 2006; Marian and Shook, 2012; Jonak, 2015). Bilingualism has many faces. For example, if the focus is on the age of acquisition and the child is adequately exposed to his/her languages since birth, then (s)he can be considered a simultaneous bilingual. If (s)he is exposed to a second language after the second/third year, then (s)he is an early or late sequential bilingual depending on whether L2 acquisition begins in early or late childhood/adulthood (De Houwer, 2009; Meisel, 2009; Paradis et al., 2011). According to the level of proficiency in their two languages, bilinguals can be dominant (if they are more fluent in one language than the other) or balanced (if they master the languages with the same proficiency) (e.g., Peal and Lambert, 1962). In this article we will refer to a pragmatic definition of bilingualism, namely the regular use of two (or more) languages independently from other variables such as age of acquisition or proficiency level (Grosjean, 1992; Marini et al., 2012; Uljarević et al., 2016).

A particularly delicate issue regards the identification of children who are exposed to different languages and have developmental language disorders (DLDs) (Kay-Raining Bird et al., 2016). The label DLDs has recently been endorsed for referral to children who have language disorders not associated with a known medical etiology even if they may co-occur with other neurodevelopmental disorders (Snowling et al., 2017). It replaces previous labels such as Specific or Primary Language Impairments. That said, the condition of bilingualism poses serious difficulties to clinicians. Children with typical language development (TLD) exposed to two or more languages may be incorrectly diagnosed with language impairments. However, individuals with DLDs exposed to a bilingual context may not be correctly diagnosed. The former error is often referred to as overdiagnosis or mistaken identity; the latter as underdiagnosis or missed identity (Salameh et al., 2002; Genesee et al., 2004; De Jong, 2008; Armon-Lotem, 2012; Grimm and Schulz, 2014). Both over- and underdiagnosis stem from the observation that children with typical development exposed to two or more languages might experience difficulties (e.g., limited verb accuracy, reduced lexical repertoire, and morphosyntactic impairments; Michael and Gollan, 2005; Bialystok et al., 2010; Vender et al., 2016) that resemble those observed in children with DLDs (e.g., Paradis and Crago, 2000; Håkansson, 2001; Paradis, 2005; Paradis et al., 2008). However, converging evidence suggests that in children with typical development who have been adequately exposed to two languages the milestones of both first and second language development may be similar to those observed in monolingual peers (e.g., Paradis et al., 2011; Marini et al., 2016). Indeed, the exposure to a bilingual context is not a risk factor for lexical development (e.g., Marini et al., 2017). Differences in the age of first exposure to the L2 (simultaneous, early or late sequential), acquisition contexts (e.g., at home or at school), and degree of exposure to both L1 and L2 might significantly affect linguistic development (Schwartz, 2004; Meisel, 2007; Paradis et al., 2010; Armon-Lotem, 2012; Cattani et al., 2014). A further complication relates to the additional effects potentially exerted by other cognitive functions. For example, increasing evidence suggests that phonological short-term and working memory play a major role in language learning and processing (e.g., Kormos and Sáfár, 2008; Engel de Abreu and Gathercole, 2012; Verhagen and Leseman, 2016; Riva et al., 2017) and significantly affect children’s performance on lexical and grammatical production tasks (Marini et al., 2014). This supports the need to consider both environmental and cognitive factors when dealing with children exposed to more than one language. Only such a comprehensive account of the child’s bilingual experience and cognitive profile may allow clinicians to correctly interpret their performance on standardized linguistic testing. Crucially, such preliminary information is necessary but not sufficient. An accurate assessment of a bilingual’s two languages requires the administration of equivalent tests in the child’s two languages with norm-referenced measures for each language across the different levels of linguistic processing (Kohnert, 2010; Jonak, 2015). The adaptation process should consider the specific characteristics of the considered languages (e.g., phonology, lexical frequencies, derivational and/or inflectional morphology, etc…) in order to ensure an accurate comparison of the linguistic competence of the children across their two languages.

The majority of the studies focusing on bilinguals with DLDs have not included all the information needed to correctly evaluate the participants’ linguistic performance. Nonetheless, the available evidence highlights that the exposure to a bilingual context does not complicate the process of linguistic acquisition in children with DLDs (Kohnert, 2010). Simultaneous and early sequential bilinguals with DLDs tend to have similar patterns of impairment as monolinguals with DLDs (Håkansson et al., 2003; Paradis et al., 2003, 2006; Rothweiler et al., 2012). For example, in Paradis et al. (2003) three groups of 7-year-old children with DLDs (monolingual English- and French-speaking and simultaneous bilinguals speaking both French and English) produced similar morphosyntactic errors on a linguistic production task. Namely, all of them produced more errors (and had similar accuracy levels) while using morphemes carrying information about verbal tense than in the use of the other inflective morphemes. Similarly, Rothweiler et al. (2012) investigated verbal morphology in German in 6 monolingual German-speaking children with DLDs, 6 early sequential bilinguals with DLDs (Turkish L1 and German L2), and 6 early sequential bilinguals with TLD (Turkish L1 and German L2). The bilinguals had been first exposed to their L2 between 2;09 and 4;04 years. Impairments in Subject-Verb agreement in German were similar for bilingual and monolingual children with DLDs suggesting that such morphosyntactic difficulties in subject-verb agreement might reflect a clinical marker of DLDs in German for both mono- and bilinguals. This finding was supported also by a following study by Clahsen et al. (2014), where the same 18 participants as in Rothweiler et al. (2012) did not differ in another morphosyntactic feature of German language, i.e., past participle inflection. Even if they did not control for the potential impact of phonological short-term and working memory on the morphological difficulties of these children, studies such as those by Rothweiler et al. (2012) are particularly interesting as they include a comparison between bilinguals with and without DLDs (see also Gutiérrez-Clellen and Simon-Cereijido, 2007). Indeed, as even children with typical development might experience difficulties in their L2s, comparing their L2 linguistic (i.e., lexical and grammatical) performance with that of bilingual children with DLDs may be misleading from a clinical point of view suggesting the presence of a potential impairment in typically developing L2 learners (e.g., Windsor and Kohnert, 2004).

Bilingual children with DLDs have difficulties in both of their languages (Restrepo and Kruth, 2000; Peña et al., 2001; Cleave et al., 2010; Kohnert, 2010; Marini et al., 2012; but see Jacobson and Livert, 2010) and learn them at a slower pace than bilingual peers with typical development (Håkansson et al., 2003). Over the past 10 years, some studies have begun to investigate the linguistic performance of bilingual children with DLDs also by using procedures of narrative assessment (Cleave et al., 2010; Squires et al., 2014; Rezzonico et al., 2015). These have proved particularly informative with respect to traditional standardized testing. For example, Cleave et al. (2010) compared the narrative skills in English of 14 monolinguals and 12 bilinguals with DLDs (who had English as L1 and different languages as L2). Based on parent reports, the bilinguals were exposed to their L2 in the home for at least 25% and spoke that language at least 10% of their daily time. Unfortunately, the two groups were not balanced for Socio-Economic Status and monolinguals had parents with higher levels of education. For this reason, the authors used maternal education level as a covariate in their analyses. On two tasks assessing expressive morphosyntax [i.e., the word structure subtest of the Clinical Evaluation of Language Fundamentals-Preschool 2 (CELF-P2; Wiig et al., 2004) and the Structured Photographic Expressive Language Test – Preschool 2 (SPELT-P2; Dawson et al., 2005)] monolingual children with DLDs outperformed bilinguals. However, when asked to perform a narrative story production task and a story retelling task, the two groups of children were no longer different in any narrative or linguistic measure. Most importantly, they had similar difficulties also on morphological and grammatical measures. Unfortunately, in this study the children’s performance on the two narrative production tasks was presented as a composite score. This does not allow readers to discern whether one of these two tasks is more informative than the other and their respective impact on the composite indexes used for the analyses. This is a significant *vulnus* in the literature as story retelling and story generation tasks exert different cognitive and linguistic demands on children. The former rely heavily on memory, whereas the latter on executive (i.e., phonological short-term and working memory, inhibition, monitoring and planning) and discourse selection and organization skills. As to this regard, Marini et al. (2012) compared the linguistic skills of nine bilingual children with DLDs aged between 6 and 13 years and exposed to Friulian L1 and Italian L2. The assessment focused on lexical comprehension, sentence comprehension, sentence repetition, semantic fluency, and the ability to produce a story on a picture-description task. In this study almost all participants experienced similar difficulties across the two languages. Most interestingly, the picture descriptions had similar levels of productivity (in terms of words and mean length of utterance), contained similar amounts of different words (i.e., types) and similar percentages of phonological semantic and morphological errors across the two languages.

In summary, the available evidence suggests that in order to adequately describe the linguistic profile of bilingual children with DLDs it is necessary to gather a comprehensive amount of information about their bilingual history and cognitive profile. Furthermore, their linguistic skills should be analyzed by using equivalent tests with the inclusion of narrative production tasks. This pilot study has been designed to identify the linguistic characteristics of a cohort of 11 simultaneous and early bilingual children (Italian, L1; German L2) with DLDs in each of their languages by using equivalent forms of the same battery of tests which includes also a narrative generation task (the *Battery for the assessment of language in children aged 4 to 12* – BVL_4-12; Marini et al., 2015) and comparing their performance with those of bilingual children with TLD. The two groups were matched for a number of environmental variables (i.e., language exposure, socio-economical status of their families, level of reading in families, etc…) that are known to exert a significant impact on language development. As children with DLDs usually have difficulties in phonological short-term and working memory (e.g., Vugs et al., 2014) that might affect their linguistic performance (Marini et al., 2014), we included in the assessment also a task aimed at controlling for this potentially confounding variable. We hypothesized that (1) the participants with typical development would outperform the bilinguals with DLDs on phonological short-term memory; (2) phonological short-term memory would be significantly correlated with measures of lexical and grammatical production (Marini et al., 2014); (3) after controlling for the effect of phonological short-term memory children with typical development would have higher scores on tasks assessing lexical and grammatical skills; and (4) children with DLDs would have similar skills in both languages.

## Materials and Methods

### Participants

Twenty-two Italian-German bilingual children were included in this study. Eleven participants had previously received a diagnosis of DLDs using standardized testing in both languages in rehabilitation centers in Bolzano’s area. The remaining eleven children had typical cognitive and language development (TLD). All participants came from families living in Bolzano’s area, a bilingual region in north-eastern Italy where both Italian and a variety of German (i.e., the Austrian-Bavarian dialect) are currently spoken. Children with TLD were recruited in mainstream schools, whereas children with DLDs were recruited in rehabilitation centers. Parents provided family demographic information and children’s daily language usage and exposure at home through a questionnaire. Importantly, all the participants had been consistently exposed also to Standard German at school since the age of 3 years. For all children both parents released their written and informed consent to the participation of their children to the study and to data processing. The study was approved by the Ethical Committee of the Hospital of Bolzano.

As shown in Table 1, no group-related differences were found for chronological age [*t*(20) = 0.416; *p* = 0.682], level of formal education in both Italian and German [χ^2^_(3,_ *_N_*_=__22)_ = 3.818; *p* = 0.282], gender distribution [χ^2^_(1,_ *_N_*_=__22)_ = 0.000; *p* = 1.000], and handedness [χ^2^_(2,_ *_N_*_=__22)_ = 0.500; *p* = 0.458]. Furthermore, the two groups were balanced also for a number of other variables that have been shown to exert a significant effect on language development: the levels of exposure to the two languages [χ^2^_(2,_ *_N_*_=__22)_ = 2.111; *p* = 0.348], parental education [χ^2^_(1,_ *_N_*_=__22)_ = 4.070; *p* = 0.131], and the number of books averagely read in each family [χ^2^_(1,_ *_N_*_=__22)_ = 3.619; *p* = 0.460]. As mentioned, the two groups had similar levels of exposure to both Italian and German: they did not differ on the languages used at home [χ^2^_(2,_ *_N_*_=__22)_ = 0.220; *p* = 0.896], the language used by the fathers or mothers to communicate with their children [χ^2^_(2,_ *_N_*_=__22)_ = 2.392; *p* = 0.302, and χ^2^_(2,_ *_N_*_=__22)_ = 1.200; *p* = 0.549, respectively], as well as the language used by the children to talk to their fathers [χ^2^_(2,_ *_N_*_=__22)_ = 0.667; *p* = 0.717] and mothers [χ^2^_(2,_ *_N_*_=__22)_ = 1.200; *p* = 0.549]. Finally, the two groups did not differ on the Raven’s progressive matrices (Raven, 1938) [*t*(20) = 1.586; *p* = 0.129] but differed on a task of phonological short-term memory, i.e., the forward digit recall subtest of the Wechsler Scales (Wechsler, 1993) [*t*(20) = 3.210; *p* < 0.004] (See Table 1).

**Table 1:** Means, standard deviations, ranges, and percentages showing demographic data of the two groups of participants and their performance at the non-verbal reasoning and phonological short-term memory tasks.

General Information	TLDs (*N* = 11)			DLDs (*N* = 11)		
	*M*	(*SD*)	Min–Max	*M*	(*SD*)	Min–Max
Age	8.79	(0.78)	[8.02–10.06]	8.59	(1.35)	[7.00–10.03]
Raven’s Matrices (raw)	30.46	(2.54)	[34–41]	28.36	(3.56)	[21–36]
Digit Span Forward (span)^∗^	5.64	(0.81)	[5–7]	4.64	(0.81)	[4–6]

	**Frequency**		**Percent**	**Frequency**		**Percent**

**Gender**						
Males	6		55	6		55
Females	5		45	5		45
**Manual dominance**						
Right	11		100	9		82
Left	0		0	2		18
**Parents’ education**						
High	5		45	2		18
Low	6		55	9		82
**Reading in Families (number of books)**						
<80	5		46	8		73
>80	6		54	3		27
**The children are more exposed to**						
Italian	3		27	1		9
German	5		46	4		36
Both	3		27	6		55
**The father talks to his child in**						
Italian	8		73	9		82
German	1		9	2		18
Both	2		18	0		0
**The child answers to his/her father in**						
Italian	8		73	8		73
German	1		9	2		18
Both	2		18	1		9
**The mother talks to her child in**						
Italian	2		18	3		27
German	8		73	8		73
Both	1		9	0		0
**The child answers to his/her mother in**						
Italian	2		18	3		27
German	8		73	8		73
Both	1		9	0		0


*Asterisks show when a group-related difference was significant. Legend: DLDs, children with Developmental Language Disorders; TLD, children with Typical Language Development.*

### Procedures of Linguistic Assessment

The participants’ linguistic skills in the two languages were assessed by administering the Italian and the German versions of the *Batteria per la Valutazione del Linguaggio in bambini dai 4 ai 12 anni* (*Battery for the assessment of language in children aged 4 to 12* – BVL_4-12; Marini et al., 2015). The BVL_4-12 has been designed to assess production, comprehension, and repetition abilities in children aged 4 through 12 years. The Italian version of the BVL_4-12 has been recently standardized in Italian. This Battery is currently under adaptation and standardization in Slovenian, Spanish, Russian, and German. For the adaptation to German particular care was paid to the selection of language-specific items for each task (i.e., by considering lexical frequencies as well as the grammatical characteristics of German). The order of language administration (Italian vs. German) was randomized across subjects. We will report the performance of these children only on tasks assessing lexical, grammatical and discourse skills.

### Assessment of Lexical Skills

The participants’ lexical abilities were assessed by administering the naming, discourse production, and lexical comprehension subtests of the BVL_4-12.

Their ability to select and produce words in German and Italian was explored by administering a naming and a discourse production task. In the former children were asked to name a series of black and white pictures (67 for Italian and 67 for German). For each language, the images elicited a maximum of 67 correct answers that had been controlled for frequency of use and semantic category. In order to further control for the adequacy of this adapted task, the participants were also administered a naming task which has been already standardized in German, i.e., the naming task of the Neuropsychologisches Screening für 5-11-jährige Kinder (Kaufmann et al., 2008). Additional information about the participants’ lexical production skills were obtained by administering a discourse production task where children were asked to describe the story portrayed in one vignette made of six pictures provided in the correct order on the same page (the ”Nest Story” by Paradis, 1987). Each narrative sample was recorded and transcribed. The transcriptions included potential phonological fillers, pauses, false starts, phonetic/phonological errors, neologisms, and extraneous utterances. Two independent raters analyzed the transcripts following the multilevel analysis of discourse production outlined in Marini et al. (2011). Acceptable inter-rater reliability was set at Cohen’s *k* ≥ 0.80. Namely, lexical production skills in each language were assessed by calculating a percentage of semantic paraphasias, i.e., words that replaced semantically related target words (e.g., “*flower*” in the sentence “They are looking at the flower,” where the speaker meant “*tree*”). The percentage of Semantic Paraphasias was calculated by using the following formula: [(semantic paraphasias/words)^∗^100]. The participants’ lexical comprehension skills were assessed by administering the Italian and German versions of the lexical comprehension task of the BVL_4-12. They were asked to identify which, among four pictures, best represented the meaning of a series of 41 target words uttered by the examiner. The target words were selected according to their frequency of occurrence in German and Italian (i.e., high, medium, and low), respectively. One of the pictures represented the target word (e.g., “cat”), whereas the remaining three pictures portrayed a semantic (e.g., “dog”), a phonological (e.g., “car”), and an unrelated distractor (e.g., “table”). Children received 1 point for each correct answer. The maximum score for lexical comprehension was 41 for each language.

### Assessment of Grammatical Skills

The participants’ grammatical comprehension abilities were investigated with the German and Italian versions of the grammatical comprehension task of the BVL_4-12. This evaluates the ability of the children to understand sentences with varying syntactic complexity. After hearing each stimulus sentence (e.g., “the girl pushes the boy”), children were showed a sheet with four pictures: one represented the meaning of the target sentence whereas the remaining three pictures represented grammatical distractors (e.g., “the girl pushes the boys,” “the girls push the boy,” or “the girls push the boys,” respectively). For each correct answer, the children received 1 point with a maximum score of 40 for each language. In order to further control for the adequacy of this adapted task, the participants were also administered a Grammatical Comprehension task which has been already standardized in German, i.e., the one in the Neuropsychologisches Screening für 5-11-jährige Kinder (Kaufmann et al., 2008).

The participants’ grammatical production skills were determined by calculating a percentage of paragrammatic errors and one of complete sentences produced during the generation of the Nest Story (Marini et al., 2011). Paragrammatic errors are scored whenever a child replaces a correct function word or bound morpheme with a wrong one. The percentage of paragrammatic errors was calculated by dividing the number of such errors by the number of utterances {i.e., [(Paragrammatic errors/utterances)^∗^100]}. The percentage of Complete Sentences was derived by dividing the number of grammatically complete sentences by the utterances {i.e., [(complete sentences/utterances)^∗^100]}. Utterances were identified according to the criteria outlined in Marini et al. (2011). A sentence was considered complete if it contained all the arguments necessarily required by the verb and no omissions or substitutions of free or bound morphemes.

### Assessment of Discourse Skills

The macrolinguistic analysis included two measures of discourse organization (i.e., percentages of errors of local and global coherence) and one of informative content (percentage of lexical informativeness). The % of Local Coherence Errors measured the extent to which each utterance was conceptually related to the preceding one and included both missing referents and topic shifts. Here the notion of topic shift is used in its general meaning. Consequently, topics shift were scored whenever an utterance was interrupted and the following one introduced a new topic without completing the one left suspended as in the following example: “/ *They are going to … / the man is on the ground /*.” A missing referent was identified whenever the referents in a sentence were ambiguous or incorrect. The % of Local Coherence Errors was calculated as follows: [(local coherence errors/utterances)^∗^100].

Global coherence errors include the production of utterances that: (1) repeat previously introduced concepts (repetitive utterances, as the underlined utterance in the following example: /the woman asks him to take the bird / she wants him to take it /); (2) do not provide any additional information (filler utterances; as the underlined utterances in the following example: /they are calling him / what else? / let me think /); (3) derail from the flow of discourse (tangential utterances; as in /They are under a tree/ This tree looks like the one I have in my garden / In spring it will be full of flowers/); (4) include ideas that are conceptually incongruent with the stimulus (conceptually incongruent utterances; as in /He is going up there / a neighbor calls him / [in the story there is no neighbor calling him]). The percentage of global coherence errors was calculated as follows: [(global coherence errors/utterances)^∗^100].

The informative content of each storytelling was calculated in terms of a percentage of Lexical Information Units (LIUs) to words [(lexical information units/words)^∗^100]. LIUs included those words that were appropriately used in the text (Marini and Urgesi, 2012). Those words that had been scored as errors of any kind (including words embedded in filler, repeated, incongruent or tangential utterances) were excluded from the count of LIUs.

## Results

### Analysis of Lexical Skills

A group-related difference was found at the Forward Digit Span task. For this reason, the presence of differences on lexical measures (i.e., naming, % semantic errors, and lexical comprehension) was investigated considering the potentially confounding role of phonological short-term memory (see Table 2). The relationship between performance at the Forward Digit Span task and the measures tapping lexical skills was investigated using Pearson product-moment correlation coefficient on the whole sample of participants after summing the scores obtained in the two languages. A significant positive correlation was found only between forward digit span and naming (*r* = 0.640; *p* < 0.001). For this reason, the group-related difference on naming was analyzed by performing one repeated measure ANCOVA with group (i.e., DLDs vs. TLD) as between-subject factor, language (Italian vs. German) as within-subject factor, the participants’ performance at the Forward Digit Span task as covariate, and the naming scores as dependent variables. For the remaining two variables no covariation was needed and two repeated measures’ ANOVAs with group (i.e., DLDs vs. TLD) as between-subject factor and language (Italian vs. German) as within-subject factor and the % semantic errors and lexical comprehension as dependent variables were performed. The *alpha* level was set at *p* < 0.017 (0.05/3 dependent variables) after Bonferroni correction for multiple comparisons.

**Table 2:** Results (means and SDs) of the assessment of lexical skills in the two groups of participants.

Lexical skills	TLDs			DLDs		
	*M*	(*SD*)	[Min–Max]	*M*	(*SD*)	[Min–Max]
**Naming^∗^**						
Italian	61.27	(3.41)	[56–66]	54.00	(7.78)	[39–65]
German	61.09	(4.11)	[53–67]	52.73	(6.75)	[43–61]
**% Semantic Paraphasias^∗^**						
Italian	1.69	(3.16)	[0–10]	2.60	(2.30)	[0–6]
German	1.47	(2.17)	[0–6]	5.91	(5.71)	[0–17]
**Lexical Comprehension^∗^**						
Italian	34.27	(4.08)	[27–41]	32.09	(4.32)	[24–38]
German	37.91	(1.64)	[35–41]	33.64	(3.61)	[28–39]


*Asterisks show when a group-related difference was significant after Bonferroni correction for multiple comparisons. Legend: DLDs, children with Developmental Language Disorders; TLD, children with Typical Language Development.*

Children with DLDs performed worse than controls on the naming tasks [*F*(1,19) = 10.818; *p* < 0.004; partial η^2^ = 0.363]. Furthermore, they produced more semantic errors [*F*(1,20) = 7.315; *p* < 0.014; partial η^2^ = 0.268] and understood fewer words on the lexical comprehension subtest of the BVL_4-12 [*F*(1,20) = 14.985; *p* < 0.001; partial η^2^ = 0.428]. Importantly, no within-subject effects were found for any of these three measures (*p* = 0.529, *p* = 0.210, and *p* = 0.056, respectively).

### Analysis of Grammatical Skills

Among the measures assessing grammatical skills (i.e., % paragrammatic errors, % complete sentences and grammatical comprehension; see Table 3), only the % of complete sentences correlated significantly with Forward Digit Span task (*r* = 0.542; *p* < 0.009). For this reason, the group-related difference on this variable was analyzed by performing one repeated measures ANCOVA with group (i.e., DLDs vs. TLD) as between-subject factor, language (Italian vs. German) as within-subject factor, and the participants’ performance at the Forward Digit Span task as covariate. For the remaining two variables no covariation was needed and two repeated measures’ ANOVAs with group (i.e., DLDs vs. TLD) as between-subject factor and language (Italian vs. German) as within-subject factor and the % of Paragrammatic errors and the score at the grammatical comprehension task as dependent variables were performed. *Alpha* level was set at *p* < 0.017 (0.05/3 dependent variables) after Bonferroni correction.

**Table 3:** Results (means and SDs) of the assessment of grammatical skills in the two groups of participants.

Morphosyntactic skills	TLDs			DLDs		
	*M*	*(SD)*	[Min–Max]	*M*	(*SD*)	[Min–Max]
**% Paragrammatisms**^§^						
Italian	1.68	(1.49)	[0–4]	2.49	(1.75)	[0–5]
German	3.88	(3.59)	[0–9]	4.99	(2.44)	[1–8]
**% Complete Sentences**						
Italian	59.59	(23.47)	[29–100]	55.84	(21.07)	[19–94]
German	58.36	(14.59)	[36–83]	45.44	(20.29)	[17–75]
**Grammatical Comprehension**						
Italian	37.36	(2.11)	[33–40]	36.27	(3.47)	[27–39]
German	37.55	(2.46)	[32–40]	35.09	(3.21)	[30–39]


*^§^Shows when the language difference was significant. Legend: DLDs, children with Developmental Language Disorders; TLD, children with Typical Language Development.*

No group-related difference was found in the % of paragrammatic errors [*F*(1,20) = 1.671; *p* = 0.211; partial η^2^ = 0.077] with a significant within-subject effect (*p* < 0.005) which, however, did not determine a significant Language ^∗^ Group interaction (*p* = 0.839). No group-related differences were found for the % of complete sentences [*F*(1,20) = 3.131; *p* = 0.092; partial η^2^ = 0.135] with no significant within-subject effect (*p* = 0.427). Finally, no group-related difference was found for grammatical comprehension [*F*(1,19) = 0.789; *p* = 0.385; partial η^2^ = 0.040], again with no significant within-subject effect (*p* = 0.484).

### Analysis of Discourse Production Skills

As no significant correlation was found between the scores at the forward digit span and discourse production (% errors of local coherence, % errors of global coherence, % lexical informativeness), group-related differences in these measures were investigated with three repeated measures’ ANOVAs with group (i.e., DLDs vs. TLD) as between-subject factor, language (Italian vs. German) as within-subject factor and the three narrative measures as dependent variables (see Table 4). *Alpha* level was set at *p* < 0.017 (0.05/3 dependent variables) after Bonferroni correction.

**Table 4:** Results (means and SDs) of the assessment of discourse skills in the two groups of participants.

Narrative skills	TLDs			DLDs		
	*M*	(*SD*)	[Min–Max]	*M*	(*SD*)	[Min–Max]
**% Errors of Local Coherence^∗^**						
Italian	24.38	(20.06)	[0–60]	39.66	(21.02)	[7–75]
German	25.35	(14.79)	[8–50]	53.71	(13.73)	[32–70]
**% Errors of Global Coherence**						
Italian	8.19	(8.23)	[0–25]	9.92	(9.31)	[0–25]
German	4.77	(11.64)	[0–39]	13.93	(10.49)	[0–29]
**% Lexical Informativeness^∗^**						
Italian	85.57	(9.26)	[67–98]	83.27	(5.99)	[73–94]
German	84.73	(13.06)	[50–100]	69.13	(13.69)	[44–83]


*The asterisks show when the group-related difference was significant. Legend: DLDs, children with Developmental Language Disorders; TLD, children with Typical Language Development.*

Children with DLDs produced more violations of local coherence than controls [*F*(1,20) = 22.557; *p* < 0.001; partial η^2^ = 0.530] with no within-subject effect (*p* = 0.224). On the contrary, the two groups did not differ on the production of errors of global coherence [*F*(1,20) = 4.568; *p* < 0.045; partial η^2^ = 0.186], again with no significant within-subject effect (*p* = 0.932). Finally, participants with DLDs produced significantly fewer informative words than controls [*F*(1,20) = 10.895; *p* < 0.004; partial η^2^ = 0.353] with no within-subject effect (*p* = 0.063).

### Further Inspection of the Reliability of the Adapted Tasks

In order to control for the reliability of the adapted versions of the naming and the grammatical comprehension subtests of the BVL_4-12, the participants received also two tasks assessing the same skills that have already been standardized in German. These are the naming and the grammatical comprehension subtests of the Neuropsychologisches Screening für 5-11-jährige Kinder (Kaufmann et al., 2008). The relationship between performance at the Naming and Grammatical comprehension tasks in German of the BVL_4-12 and of the Neuropsychologisches Screening für 5-11-jährige Kinder was investigated using Pearson product-moment correlation coefficient. This analysis showed significant robust correlations between the two naming (*r* = 0.810; *p* < 0.001) and grammatical comprehension tasks (*r* = 0.758; *p* < 0.001).

## Discussion

In the current pilot study we investigated language skills in a cohort of simultaneous bilingual Italian-German speaking children with and without developmental language impairments. The participants were balanced for a number of environmental variables that are known to excert a major influence on bilingual development. Their linguistic performance (in terms of lexical, grammatical, and narrative abilities) was assessed by administering a selection of the subtests of the BVL 4-12 (Marini et al., 2015) and its adapted version to German. Overall, the results showed that: the participants with DLDs had reduced phonological short-term memory (confirmation of Hypothesis 1); that phonological memory correlated with Naming and % of Complete Sentences (confirmation of Hypothesis 2); that children with DLDs had reduced lexical skills that, in turn, likely contributed to the reduced levels of local coherence and informativeness of their narratives (partial confirmation of Hypothesis 3); and that these difficulties were found at similar levels in their two languages (in line with previous findings by Restrepo and Kruth, 2000; Peña et al., 2001; Cleave et al., 2010; Kohnert, 2010; Marini et al., 2012). The only exception was the production of paragrammatic errors that were language dependent in both groups (partial confirmation of Hypothesis 4). A final consideration concerns the significant positive correlations observed for the Naming and Grammatical comprehension tasks in German of the BVL_4-12 and the same tasks of the Neuropsychologisches Screening für 5-11-jährige Kinder, suggesting the reliability of the adapted versions of these two tasks.

As a first point, this study confirms previous investigations reporting phonological short-term memory difficulties in monolingual (e.g., Ellis Weismer et al., 1999; Montgomery, 2006; Marini et al., 2014) and sequential bilingual children with DLDs (Engel de Abreu et al., 2014) and extends this observation also to simultaneous bilinguals. It supports the possibility that a reduced phonological short-term memory is a characteristic feature of all children with DLDs (including both mono- and bilinguals). According to the phonological storage deficit hypothesis, difficulties in keeping track of phonological and/or lexical items in short-term memory and eventually process them in working memory might result in slowed vocabulary acquisition (e.g., Gathercole and Baddeley, 1990; Marini et al., 2017) and affect their linguistic development (Bishop, 2006; Archibald and Gathercole, 2007; Graf Estes et al., 2007). In line with this hypothesis, phonological short-term memory correlated with the performance on a task assessing lexical selection in both languages (i.e., Naming) and the % Complete Sentences derived by the multilevel analysis of discourse which assesses the ability to generate complete sentences during the production of a narrative discourse. The specific contributions of phonological short-term memory on these linguistic skills will be discussed later.

The cohort of children with DLDs produced fewer correct lexical items on the naming task, more semantic errors on the narrative production task and understood fewer words on the lexical comprehension test. Such findings suggest that in this cohort of simultaneous bilinguals with DLDs lexical selection is an area of major weakness. According to an influential model of message production (e.g., Levelt, 1989; Levelt et al., 1999; Indefrey and Levelt, 2000), during the process of lexical selection, the target word is activated also because of the co-occurring inhibition of semantically related competitors. In this process, not only short-term memory but also additional executive skills such as inhibition, attention and planning play a critical role. In a recent investigation, a group of 15 Portuguese–Luxembourgish bilinguals from Luxembourg with a diagnosis of DLDs did not manifest the same advantages in selective attention and interference suppression as 33 typically developing Portuguese–Luxembourgish bilingual peers living in Luxembourg. However, they did not lag behind another group of 33 typically developing Portuguese-speaking monolinguals from Portugal in these domains of executive functioning (Engel de Abreu et al., 2014). Based on our findings and those by Engel de Abreu et al. (2014), we hypothesize that the process of lexical selection is not affected by the condition of bilingualism (as the controls were also simultaneous bilinguals), the specific language in use (as such skills were similarly impaired in both languages), or other cognitive limitations (as the group-related differences survived the covariation for phonological short-term memory and were significant also in two measures that did not correlate with this cognitive variable). Rather, the reduced lexical selection skills may reflect a core symptom of DLDs. Indeed, the production of semantic errors is usually interpreted as a failure in this process: the selection of a semantically wrong word is likely related to a difficulty in selecting the word that best matches with the non-verbal concept selected in the previous phase of prelinguistic conceptual message formulation. Importantly, the lexical difficulties observed in the group of participants with DLDs were also related to macrolinguistic difficulties, as highlighted by the production of a number of words with no clear referents that reduced the levels of both local coherence (see also Rezzonico et al., 2015) and lexical informativeness. This further supports the need to extend the linguistic analysis also to macrolinguistic aspects of language processing in children with DLDs using discourse generation tasks (see also Cleave et al., 2010; Marini et al., 2014).

The participants’ grammatical skills were assessed in terms of production of paragrammatic (i.e., morphologic and morphosyntactic) errors, percentage of complete sentences produced in the narrative generation task, and grammatical comprehension. Interestingly, the participants with DLDs did not have more difficulties than bilingual controls on any of these three measures. Indeed, they produced the same amount of paragrammatic errors and percentage of complete sentences to utterances in the narrative production task and understood the same number of sentences in the grammatical comprehension task. Apparently, these results are at odds with those of previous investigations where impairments in inflectional morphology and morphosyntax have been considered as core symptoms of DLDs in both monolingual (e.g., Rice, 2003) and bilingual children (Jacobson and Livert, 2010; Rothweiler et al., 2012). For example, a previous study by Rothweiler et al. (2012) suggested that Subject-Verb agreement in German may be impaired in both monolinguals and early sequential bilinguals with DLDs and that such morphosyntactic difficulties might reflect a clinical marker of DLDs in German. However, our findings are coherent with previous observations showing that grammatical morphology is not necessarily a core feature of DLDs (Thordardottir, 2016). Furthermore, there is evidence that in narrative discourse production and retelling tasks such skills may not appear deficitarian (e.g., Cleave et al., 2010). In line with what reported in Cleave et al. (2010) we hypothesize that the absence of significant differences in morphosyntactic skills might be related to the use of different ways to assess such skills. Indeed, as reported also in other investigations, narrative production tasks are more adequate than decontextualized tasks to describe the actual linguistic profile of persons with DLDs (e.g., Marini et al., 2008). An alternative possibility is that the two groups did not differ on such skills as even children with typical development might experience difficulties in their L2s. Therefore, comparing their L2 grammatical performance with that of bilingual children with DLDs may be misleading from a clinical point of view suggesting the presence of a potential difficulty in typically developing L2 learners (e.g., Windsor and Kohnert, 2004). The lack of groups of monolinguals is among the limitations of this study as this omission did not allow us to determine whether the group of bilingual participants with TLD had similar grammatical skills as those of Italian-speaking or German-speaking monolinguals with TLD. Another interesting finding concerns the language-related effect found in the production of Paragrammatic errors. As already commented, the children with DLDs and those with typical development produced a similar amount of such morphological and morphosyntactic errors. However, both groups produced more such errors in German than in Italian. This further highlights the need to have equivalent tests adapted (and not simply translated) in the languages spoken by bilingual children.

Discourse production skills were assessed in terms of percentages of errors of both local and global coherence and lexical informativeness. Participants with DLDs were less informative (for a similar finding on a story retelling task see Rezzonico et al., 2015) and had more difficulties than controls in establishing local coherence among the produced utterances. On the contrary, no group-related differences were found in the production of errors of global coherence. The latter was quite expected as difficulties of global coherence likely reflect planning and monitoring difficulties that are usually observed in patients with different disorders such as Williams Syndrome (Marini et al., 2010) or Autism Spectrum Disorders (Ferretti et al., 2018; Marini et al., 2019). These findings further support the possibility that the two languages were similarly processed also at the macrolinguistic level in both groups. Importantly, a qualitative inspection of their local coherence errors showed that they did not produce any topic shift. Rather, as already discussed, their difficulties were likely related to an increased production of words with no clear referents. A lexical difficulty observed in children with DLDs in both languages affected their overall communicative skills confirming the presence of significant interactions between micro- (i.e., lexical and grammatical) and macrolinguistic (i.e., pragmatic and discourse) levels of processing.

The current investigation has some limitations that we would like to address here. First of all, the reduced number of participants. As a pilot study, only 11 participants per group were included. This obviously reduced the generalizability of the current results to the general population. However, we would like to stress that such *vulnus* is at least partially counterbalanced by the accurate matching of the two groups of participants on a number of environmental variables that are known to significantly affect the trajectory of language development in bilinguals. A second limitation concerns the afore-mentioned absence of cohorts of Italian-speaking and German-speaking monolinguals. Future investigations should consider the inclusion of larger cohorts of bilingual and monolingual participants. This might also allow researchers to run more accurate statistical analyses exploring the potential causality among the considered variables (e.g., phonological short-term memory and lexical skills) while also controlling for a potential bilingual effect on children with typical development.

## Conclusion

In conclusion, the results of the current study have both theoretical and clinical implications. From a theoretical point of view, they support the idea that impaired lexical selection is among the core symptoms of DLDs not only in monolinguals but also in simultaneous bilingual children. Furthermore, they suggest that such difficulty is independent of the specific language in use. Rather, it apparently affects all of the languages known by such bilinguals. From a clinical point of view, these results support the need for an accurate assessment of a bilingual’s linguistic competence that should be performed by using equivalent tests in the respective two languages. They also suggest to carefully consider, during the assessment procedure, the role of environmental (e.g., age of first exposure to the languages, degree of exposure, etc.) and cognitive (e.g., phonological short-term memory) factors and their potential repercussions on language development and processing. Finally, they support the usefulness of multilevel procedures of narrative analysis that significantly contribute to draw a comprehensive linguistic profile that will be pivotal to rehabilitation (Thordardottir, 2010; Thordardottir et al., 2015).

## Ethics Statement

This study was carried out in accordance with the recommendations of the “Comitato Etico dell’Ospedale di Bolzano”. All subjects released their written informed consent in accordance with the Declaration of Helsinki. The protocol was approved by the “Comitato Etico dell’Ospedale di Bolzano.”

## Author Contributions

AM planned the study, ran the statistical analyses and wrote the manuscript. PS and IR supervised the adaptation of the BVL_4-12 to German and supervised the recruitment of the participants. CS and FA co-supervised the recruitment procedure and supervised the administration of the tasks to the children. All authors contributed with comments to the interpretation of the results.

## Conflict of Interest Statement

The authors declare that the research was conducted in the absence of any commercial or financial relationships that could be construed as a potential conflict of interest.

## References

[B1] Archibald L. M. D. Gathercole S. E. (2007). Nonword repetition in specific language impairment: more than a phonological short-term memory deficit. *Psychon. Bull.* 14 919–924. 10.3758/BF03194122 18087960

[B2] Armon-Lotem S. (2012). Introduction: bilingual children with SLI - the nature of the problem. *Biling. Lang. Cogn.* 15 1–4. 10.1017/S1366728911000599

[B3] Baker C. (2006). *Foundations of Bilingual Education and Bilingualism*, 4th Edn Clevedon: Multilingual Matters.

[B4] Bialystok E. Luk G. Peets K. F. Yang S. (2010). Receptive vocabulary differences in monolingual and bilingual children. *Biling. Lang. Cogn.* 13 525–531. 10.1017/S1366728909990423 25750580 PMC4349351

[B5] Bishop D. V. M. (2006). What causes specific language impairment in children? *Curr. Dir. Psychol. Sci.* 15 217–221. 10.1111/j.1467-8721.2006.00439.x 19009045 PMC2582396

[B6] Cattani A. Abbot-Smith K. Farag R. Krott A. Arreckx F. Dennis I. (2014). How much exposure to English is necessary for a bilingual toddler to perform like a monolingual peer in language tests? *Int. J. Lang. Commun. Disord.* 49 649–671. 10.1111/1460-6984.12082 25039327

[B7] Clahsen H. Rothweiler M. Sterner F. Chilla S. (2014). Linguistic markers of specific language impairment in bilingual children: the case of verb morphology. *Clin. Linguist. Phon.* 28 709–721. 10.3109/02699206.2014.886726 24588469

[B8] Cleave P. L. Girolametto L. E. Chen X. Johnson C. J. (2010). Narrative abilities in monolingual and dual language learning children with specific language impairment. *J. Commun. Disord.* 43 511–522. 10.1016/j.jcomdis.2010.05.005 20579660

[B9] Dawson J. Stout C. Eyer J. Tattersall P. Fonkalsrud J. Croley K. (2005). *Structured Photographic Expressive Language Test—Preschool*, 2nd Edn DeKalb, IL: Janelle.

[B10] De Houwer A. (2009). *Bilingual First Language Acquisition.* North York: Multilingual Matters.

[B11] De Jong J. (2008). “Bilingualism and language impairment”, in *The Handbook of Clinical Linguistics*, eds Ball J.M. Perkins M.R. Müller N. Howard S. (Hoboken, NJ: Blackwell Publishing), 261–274. 10.1002/9781444301007.ch16

[B12] Ellis Weismer S. Evans J. Hesketh L. (1999). An examination of verbal working memory capacity in children with specific language impairment. *J. Speech Lang. Hear. Res.* 42 1249–1260. 10.1044/jslhr.4205.1249 10515519

[B13] Engel de Abreu P. M. J. Cruz-Santos A. Puglisi M. (2014). Specific language impairment in language-minority children from low-income families. *J. Commun. Disord.* 49 736–747. 10.1111/1460-6984.12107 25209889

[B14] Engel de Abreu P. M. J. Gathercole S. E. (2012). Executive and phonological processes in second-language acquisition. *J. Educ. Psychol.* 104 974–986. 10.1037/a0028390

[B15] Ferretti F. Adornetti I. Chiera A. Nicchiarelli S. Valeri G. Magni R. (2018). Time and Narrative: an investigation of storytelling abilities in children with Autism Spectrum Disorder. *Front. Psychol.* 9:944. 10.3389/fpsyg.2018.00944 29971024 PMC6018079

[B16] Gathercole S. Baddeley A. (1990). Phonological memory deficits in language disordered children: is there a causal connection? *J. Mem. Lang.* 29 336–360. 10.1016/0749-596X(90)90004-J

[B17] Genesee F. Paradis J. Crago M. (2004). *Dual Language Development and Disorders: A Handbook on Bilingualism and Second Language Learning.* Baltimore, MD: Brookes.

[B18] Graf Estes K. Evans J. L. Else-Quest N. M. (2007). Differences in the nonword repetition performance of children with and without specific language impairment: a meta-analysis. *J. Speech Lang. Hear. Res.* 50 177–195. 10.1044/1092-4388(2007/015) 17344558

[B19] Grimm A. Schulz P. (2014). Specific language impairment and early second language acquisition: the risk of over- and under diagnosis. *Child Indic. Res.* 7 821–841. 10.1007/s12187-013-9230-6

[B20] Grosjean F. (1992). “Another view of bilingualism”, in *Cognitive Processing in Bilinguals*, ed Harris R. J. (Amsterdam: Elsevier), 51–62. 10.1016/S0166-4115(08)61487-9

[B21] Gutiérrez-Clellen V. F. Simon-Cereijido G. (2007). The discriminant accuracy of a grammatical measure with Latino English-speaking children. *J. Speech Lang. Hear. Res.* 50 968–981. 10.1044/1092-4388(2007/068) 17675599 PMC3367477

[B22] Håkansson G. (2001). Tense morphology and verb-second in Swedish L1 children, L2 children and children with SLI. *Biling. Lang. Cogn.* 4 85–99. 10.1017/S1366728901000141

[B23] Håkansson G. Salameh E. Nettelbladt U. (2003). Measuring language development in bilingual children: Swedish-Arabic children with and without language impairment. *Linguistics* 41 255–288. 10.1515/ling.2003.009

[B24] Indefrey P. Levelt W. J. M. (2000). “The neural correlates of language production”, in *The New Cognitive Neurosciences*, ed. Gazzaniga M.S. (Cambridge, MA: MIT Press), 845–865.

[B25] Jacobson P. Livert D. (2010). English past tense use as a clinical marker in older bilingual children with language impairment. *Clin. Linguist. Phon.* 24 101–121. 10.3109/02699200903437906 20100041

[B26] Jonak J. (2015). Bilingual language development and language impairment in children. *Acta Neuropsychol.* 13 63–79.

[B27] Kaufmann L. Landerl K. Mazzoldi M. Moeller K. Pastore N. Salandin M. (2008). *Neuropsychologisches Screening für 5-11-jährige Kinder (Deutschsprachige Bearbeitung).* Göttingen: Hogrefe.

[B28] Kay-Raining Bird E. Genesee F. Verhoeven L. (2016). Bilingualism in children with developmental disorders: a narrative review. *J. Commun. Disord.* 63 1–14. 10.1016/j.jcomdis.2016.07.003 27461977

[B29] Kohnert K. (2010). Bilingual children with primary language impairment: issues, evidence and implications for clinical actions. *J. Commun. Disord.* 43 456–473. 10.1016/j.jcomdis.2010.02.002 20371080 PMC2900386

[B30] Kormos J. Sáfár A. (2008). Phonological short-term memory and foreign language performance in intensive language learning. *Biling. Lang. Cogn.* 11 261–271. 10.1017/S1366728908003416

[B31] Levelt W. J. M. (1989). *Speaking: from Intention to Articulation.* Cambridge, MA: MIT Press.

[B32] Levelt W. J. M. Roelofs A. Meyer A. S. (1999). A theory of lexical access in speech production. *Behav. Brain Sci.* 22 1–38. 10.1017/S0140525X99001776 11301520

[B33] Marian V. Shook A. (2012). The cognitive benefits of being bilingual. *Cerebrum* 13 1–12. PMC3583091 23447799

[B34] Marini A. Andreetta S. Del Tin S. Carlomagno S. (2011). A multi-level approach to the analysis of narrative language in Aphasia. *Aphasiology* 25 1372–1392. 10.1080/02687038.2011.584690

[B35] Marini A. Eliseeva N. Fabbro F. (2016). Impact of early second-language acquisition on the development of first language and verbal short-term and working memory. *Int. J. Biling. Educ. Biling.* 22 165–176. 10.1080/13670050.2016.1238865 28928681

[B36] Marini A. Ferretti F. Chiera A. Magni R. Adornetti I. Nicchiarelli S. (2019). Episodic future thinking and narrative discourse generation in children with Autism Spectrum Disorders. *J. Neurolinguistics* 49 178–188. 10.1016/j.jneuroling.2018.07.003

[B37] Marini A. Gentili C. Molteni M. Fabbro F. (2014). Differential verbal working memory effects on linguistic production in children with specific language impairment. *Res. Dev. Disabil.* 35 3534–3542. 10.1016/j.ridd.2014.08.031 25240219

[B38] Marini A. Marotta L. Bulgheroni S. Fabbro F. (2015). *Batteria per la Valutazione del Linguaggio in Bambini dai 4 ai 12 anni.* Firenze: Giunti O.S. Organizzazioni Speciali.

[B39] Marini A. Ruffino M. Sali M. E. Molteni M. (2017). The role of phonological working memory and environmental factors in lexical development in Italian-speaking late talkers: a one-year follow-up study. *J. Speech Lang. Hear. Res.* 60 3462–3473. 10.1044/2017_JSLHR-L-15-0415 29121196

[B40] Marini A. Tavano A. Fabbro F. (2008). Assessment of linguistic abilities in Italian children with Specific Language Impairment. *Neuropsychologia* 46 2816–2823. 10.1016/j.neuropsychologia.2008.05.013 18579167

[B41] Marini A. Urgesi C. Fabbro F. (2012). “Clinical neurolinguistics of bilingualism”, in *The Handbook of the Neuropsychology of Language* Vol. 2 ed. Faust M. (Hoboken, NJ: Blackwell), 738–759. 10.1002/9781118432501.ch36

[B42] Marini A. Martelli S. Gagliardi C. Fabbro F. Borgatti R. (2010). Narrative language in Williams Syndrome and its neuropsychological correlates. *J. Neurolinguistics* 23 97–111. 10.1016/j.jneuroling.2009.10.002

[B43] Marini A. Urgesi C. (2012). Please get to the point! A cortical correlate of linguistic informativeness. *J. Cogn. Neurosci.* 24 2211–2222. 10.1162/jocn_a_00283 22905815

[B44] Meisel J. (2007). The weaker language in early child bilingualism: acquiring a first language as a second language? *Appl. Psycholinguist.* 28 495–514. 10.1017/S0142716407070270

[B45] Meisel J. (2009). *First and Second Language Acquisition.* Cambridge: CUP.

[B46] Michael E. B. Gollan T. H. (2005). “Being and becoming bilingual: individual differences and consequences for language production”, in *Handbook of Bilingualism: Psycholinguistic Approaches*, eds Kroll J.F. de Groot A. M. B. (New York, NY: Oxford University Press), 389–407.

[B47] Montgomery J. W. (2006). Real-time language processing in school-age children with specific language impairment. *Int. J. Lang. Commun. Disord.* 41 275–291. 10.1080/13682820500227987 16702094

[B48] Paradis J. (2005). Grammatical morphology in children learning English as a second language: implications of similarities with specific language impairment. *Lang. Speech Hear. Serv. Sch.* 36 172–187. 10.1044/0161-1461(2005/019) 16175882

[B49] Paradis J. Crago M. (2000). Tense and temporality: similarities and differences between language-impaired and second-language children. *J. Speech Lang. Hear. Res.* 43 834–848. 10.1044/jslhr.4304.834 11386472

[B50] Paradis J. Crago M. Genesee F. (2006). Domain-general versus domain-specific accounts of specific language impairment: evidence from bilingual children’s acquisition of object pronouns. *Lang. Acquis.* 13 33–62. 10.1207/s15327817la1301_3

[B51] Paradis J. Crago M. Genesee F. Rice M. (2003). French-English bilingual children with SLI: how do they compare with their monolingual peers? *J. Speech Lang. Hear. Res*. 46 113–127. 10.1044/1092-4388(2003/009) 12647892

[B52] Paradis J. Emmerzael K. Sorenson Duncan T. (2010). Assessment of English language learners: using parent report on first language development. *J. Commun. Disord.* 43 474–497. 10.1016/j.jcomdis.2010.01.002 20304411

[B53] Paradis J. Genesee F. Crago M. B. (2011). *Dual Language Development and Disorders. A Handbook on Bilingualism and Second Language Learning*, 2nd Edn Baltimore, MD: Paul H. Brookes.

[B54] Paradis J. Rice M. L. Crago M. Marquis J. (2008). The acquisition of tense in English: distinguishing child second language from first language and specific language impairment. *Appl. Psycholinguist.* 29 689–722. 10.1017/S0142716408080296 18852844 PMC2565586

[B55] Paradis M. (1987). *Bilingual Aphasia Test.* New Jersey, NJ: Lawrence Erlbaum Associates.

[B56] Peal E. Lambert W. E. (1962). The relation of bilingualism to intelligence. *Psychol. Monogr.* 76 1–23. 10.1037/h0093840

[B57] Peña E. D. Iglesias A. Lidz C. S. (2001). Reducing test bias through dynamic assessment of children’s word learning ability. *Am. J. Speech Lang. Pathol.* 10 138–154. 10.1044/1058-0360(2001/014)

[B58] Raven J. C. (1938). *Progressive Matrices: A Perceptual Test of Intelligence.* London: H. K. Lewis.

[B59] Restrepo M. A. Kruth K. (2000). Grammatical characteristics of a Spanish-English bilingual child with specific language impairment. *Commun. Disord. Q.* 21 66–76. 10.1177/152574010002100201

[B60] Rezzonico S. Chen X. Cleave P. L. Greenberg J. Hipfner-Boucher K. Johnson C. J. (2015). Oral narratives in monolingual and bilingual preschoolers with SLI. *Int. J. Lang. Commun. Dis.* 50 830–841. 10.1111/1460-6984.12179 26215148

[B61] Rice M. L. (2003). “A unified model of specific and general language delay: grammatical tense as a clinical marker of unexpected variation”, in *Language Competence Across Populations: Toward a Definition of Specific Language Impairment*, eds Levy Y. Schaeffer J. (Mahwah, NJ: Erlbaum), 63–95.

[B62] Riva V. Cantiani C. Dionne G. Marini A. Mascheretti S. Molteni M. (2017). Working memory mediates the effects of gestational age at birth on expressive language development in children. *Neuropsychology* 31 475–485. 10.1037/neu0000376 28383969

[B63] Rothweiler M. Chilla S. Clahsen H. (2012). Subject-verb agreement in specific language impairment: a study of monolingual and bilingual German-speaking children. *Biling. Lang. Cogn.* 15 39–57. 10.3109/02699206.2014.886726 24588469

[B64] Salameh E.-K. Nettelbladt U. Håkansson G. Gullberg B. (2002). Language impairment in Swedish bilingual children: a comparison between bilingual and monolingual children in Malmö. *Acta Paediatr.* 91 229–234. 10.1111/j.1651-2227.2002.tb01700.x 11952014

[B65] Schwartz B. D. (2004). “Why child L2 acquisition?”, in *Proceedings of Generative Approaches of Language Acquisition*, eds Kampen J. van Baauw S. (Utrecht: LOT), 47–66.

[B66] Simons G. F. Fennig C. D. (2018). *Ethnologue: Languages of the World*, 21st Edn Dallas, TX: SIL International.

[B67] Snowling M. J. Thompson P. A. Greenhalgh T. The CATALISE-2 consortium (2017). Phase 2 of CATALISE: a multinational and multidisciplinary Delphi consensus study of problems with language development: terminology. *J. Child Psychol. Psychiatry* 58 1068–1080. 10.1111/jcpp.12721 28369935 PMC5638113

[B68] Squires K. E. Lugo-Neris M. J. Peña E. D. Bedore L. M. Bohman T. M. Gillam R. B. (2014). Story retelling by bilingual children with language impairments and typically developing controls. *Int. J. Lang. Commun. Disord.* 49 60–74. 10.1111/1460-6984.12044 24372886 PMC3877674

[B69] Thordardottir E. (2010). Towards evidence-based practice in language intervention for bilingual children. *J. Commun. Disord.* 43 523–537. 10.1016/j.jcomdis.2010.06.001 20655541

[B70] Thordardottir E. (2016). Grammatical morphology is not a sensitive marker of language impairment in Icelandic in children aged 4-14 years. *J. Commun. Disord.* 62 82–100. 10.1016/j.jcomdis.2016.06.001 27314205

[B71] Thordardottir E. Cloutier G. Ménard S. Pelland-Blais E. Rvachew S. (2015). Monolingual or bilingual intervention for primary language impairment? A randomized control trial. *J. Speech Lang. Hear. Res.* 58 287–300. 10.1044/2014_JSLHR-L-13-0277 25381447

[B72] Uljarević M. Katsos N. Hudry K. Gibson J. L. (2016). Practitioner review: multilingualism and neurodevelopment disorders - an overview of recent research and discussion of clinical implications. *J. Child Psychol. Psychiatry* 57 1205–1217. 10.1111/jcpp.12596 27443172

[B73] Vender M. Garraffa M. Sorace A. Guasti M. T. (2016). How early L2 children perform on Italian clinical markers of SLI: a study of clitic production and nonword repetition. *Clin. Linguist. Phon.* 30 150–169. 10.3109/02699206.2015.1120346 26810381

[B74] Verhagen J. Leseman P. (2016). How do verbal short-term memory and working memory relate to the acquisition of vocabulary and grammar? A comparison between first and second language learners. *J. Exp. Child Psychol.* 141 65–82. 10.1016/j.jecp.2015.06.015 26340756

[B75] Vugs B. Hendriks M. Cuperus J. Verhoeven L. (2014). Working memory performance and executive function behaviors in young children with SLI. *Res. Dev. Disabil.* 35 62–74. 10.1016/j.ridd.2013.10.022 24240018

[B76] Wechsler D. (1993). *WISC-R. Scala d’Intelligenza Wechsler per Bambini Riveduta.* Firenze: Giunti O.S. Organizzazioni Speciali.

[B77] Wiig E. Secord W. Semel E. (2004). *Clinical Evaluation of Language Fundamentals—Preschool*, 2nd Edn San Antonio, TX: Psychological Corporation.

[B78] Windsor J. Kohnert K. (2004). The search for common ground: part I. Lexical performance by linguistically diverse learners. *J. Speech Lang. Hear. Res.* 47 877–890. 10.1044/1092-4388(2004/065) 15324292

